# Challenges amid COVID-19 times - Review of the changing practices in a clinical chemistry laboratory from a developing country

**DOI:** 10.1016/j.amsu.2020.06.004

**Published:** 2020-06-06

**Authors:** Sibtain Ahmed, Lena Jafri, Hafsa Majid, Aysha Habib Khan, Farooq Ghani, Imran Siddiqui

**Affiliations:** Department of Pathology and Laboratory Medicine, Aga Khan University, Stadium Road, P.O. Box 3500, Karachi, 74800, Pakistan

**Keywords:** COVID-19, Laboratory, Challenges, Pakistan, Lessons

## Abstract

Corona Virus Disease 2019 (COVID-19) pandemic is the defining global health crisis of our time. Compared with its neighbors China and Iran, which were rated as epi-centers of the outbreak, Pakistan has lower standards of health care, unstable economy and dearth of financial resources to tackle the outbreak. Like other institutes and industries in the country, clinical laboratories were succumbed to a variety of challenges. This article is based on the experience and adapted workflow measures from the Clinical Chemistry laboratory at the Aga Khan University Hospital (AKUH), Karachi, which serves as a national referral center with its widespread network of satellite laboratories and phlebotomy centers across the country. It highlights the challenges faced and the appropriate responses to ensure the provision of diagnostic facilities during the COVID-19 outbreak. Furthermore, the lessons acquired and necessary preparations for the post crisis situation are also incorporated.

## Highlights

• Coronavirus disease (COVID-19) pandemic is the defining global health crisis of our time.
• Like other institutes and industries in the country, clinical laboratories were succumbed to a variety of challenges.
• The challenges the appropriate actions on ad hoc basis by a Clinical Chemistry laboratory, in Pakistan are emphasized.
• The lessons acquired and necessary preparations for the post crisis situation are also discussed.

## 1 Background

The marks of the Coronavirus Disease 2019 (COVID-19) outbreak trace back to 31^st^ December 2019, when a pneumonia of unknown etiology was informed to the world health organization (WHO) in Wuhan, China [1]. Since then the outbreak termed as a ‘pandemic’, declared by WHO on 11^th^ March 2020, has continued to cause severe morbidity and mortality in most countries globally with devastating implications [2]. Pakistan reported its first confirmed case in the metropolis of Karachi on 26^th^ February 2020, in an Iran returned traveler [3]. A point of concern for the country, was frequent exchange of travelers; with Iran and China, main reason being religious pilgrimages and business activities respectively. Consequently, by mid-March the pandemic, initially seen only in travelers coming from abroad was confirmed to have spread throughout the country with cases of local transmission and the first death on 20^th^ March 2020 [4].

Compared with its bordering neighbors China and Iran, which were rated as the initial epi-centers of the outbreak, Pakistan has lower standards of health care, unstable economy and dearth of resources to tackle the outbreak [5]. Most of the cities including the major economical hubs i.e. Karachi, Lahore and Islamabad were put on a lock down in fourth week of March to prevent the local spread with negative impact on the financial outcomes and revenue generation of most entities [6].

Health care facilities, especially tertiary care setup like the Aga Khan University Hospital (AKUH), Karachi, Pakistan, catering to COVID-19 cases, were the ones which sustained more serious blows [7]. Routine patient inflow was significantly reduced owing to the imposed lock down and psychological insecurities of visiting a facility testing and housing COVID-19 patients. Laboratories have a critical role to play during the pandemic right form diagnosis till surveillance, with the laboratory professionals as the front-line warriors [8,9]. However, unlike most laboratories in the developed countries with the essential infrastructure and financial resources, the current situation poses enormous challenges for the laboratories based in the developing world [10]. The Clinical Chemistry laboratory at the AKUH, serves as a referral center, and caters to on an average 16000 routine and specialized tests requests per day from all the provinces owing to its network of more than 250 phlebotomy stations spread across Pakistan. The laboratory was the first to be accredited by Joint Commission International Accreditation (JCIA) and the only with College of American Pathologist (CAP) accreditation in Pakistan.

From an organizational perspective, laboratory is considered a critical service in a tertiary care hospital and is expected to continue 24/7 operations even during an infectious pandemic [11]. This is also vital for the individual patient, who expects the provision of diagnostic services not to be disrupted. To ensure such continuity of services, the aim of this review is to highlight the challenges faced by a Clinical Chemistry laboratory in a developing country amid the COVID-19 crisis, the challenges, implementing changes and the lessons learnt.

### 1.1 Day to day challenges faced

#### 1.1.1 Declining test volumes

The financial ramifications of the COVID crisis were significant. Owing to the lock down across the country and social distancing campaigns, during the initial phase of the outbreak the testing volumes decreased significantly by approximately 52% of the baseline compared to pre-COVID months. This led to substantial financial decline and the revenue generation targets suffered enormously. The major reasons identified were a smaller number of subjects visiting outreach phlebotomy centers across the country, cessation of outpatient clinics and postponement of all non-emergency surgeries causing limited flow of patients to the tertiary care hospital, with which the laboratory is associated. To cope up with the situation, free of cost home sample collection was started with the aid of outreach phlebotomy centers [12]. The hospital initiated tele clinics for outpatients and door step sample collection for required diagnostic work up was propagated [13,14]. Following these measures and due to the ease in lockdown, a steady increment in volume of approximately 5% was noted at the time of publication.

#### 1.1.2 Work from home movement and remote meetings

The Clinical Chemistry faculties were divided into teams (e.g. Teams 1 and 2). This is so that, if one team needs to be quarantined, the other team could provide continuity of service [15,16]. Each team lead by a team leader consisted of three specialist pathologists and one post graduate trainee. The second team was made to work from home via Microsoft Teams and was connected with the team present in the section via sectional WhatsApp group. Within the workplace, the teams had dedicated workspaces and a distance of at least 2 m was practiced.

All the essential meetings were converted to virtual meetings via Microsoft Teams and ZOOM to ensure continuity of services and plans as laid down at the beginning of the year by the management [17,18]. In the beginning few issues pertaining to low bandwidth connection at home and lack of working knowledge of online platforms posed challenges but gradually improved.

The technical staff working at the bench side were also limited to minimize exposure. Furthermore, the staff was offered to avail their paid leaves during the outbreak. However, this does not lead to any ease for the section as majority of the staff were reluctant to go on annual earned leaves. The major reasons communicated were an apparent fear of losing the job amid the financial crisis and hindrances in execution of vacation plans which were normally considered to be undertaken during the leaves. Moreover, Pakistan, being a developing country has substantially compromised living conditions. Not unusual to find large households with an average of six to seven persons living together [19]. Many considered staying at work as a better and safer alternate than being lock downed at home. However, the staff was counselled to abide by the situation and physical presence of the staff at the laboratory was reduced by approximately 30% compared to normal by aid of modifications in duty rosters.

#### 1.1.3 Preserving jobs by cutting expenditure, salaries and compensation

As a measure to ease fixed costs and stave off job losses salaries of mid-level and senior level staff were revised with no annual increases, and pay cuts ranging from 10 to 30% were implemented. However, salaries of approximately 73% of the total staff including most junior staff and trainees was preserved to save them from excess economic burden. Furthermore, all travel, new projects and hiring were put on hold. Additionally, it was announced that the staff will further have no appraisals as scheduled for the fiscal year.

#### 1.1.4 Ensuring adequate inventory and supplies

Adequate supply of Personal Protective Equipment (PPE) as an essential requirement was ensured with the assistance of the purchase department. Staff was counselled to practice judicious use of PPE and sanitizers. Bench in charges were reinforced to re-visit their inventory requirements owing to the expected delays in shipment due to the air space closure, to maintain demand-supply chain. Despite the reduction in overall test volumes, a few tests e.g. Albumin, Troponin-I (Trop-I) and C - reactive protein (CRP) exhibited more than usual demands as they were being used for COVID-19 cases in supplementation to the molecular diagnosis and later for prognosis. Furthermore, due to rapid influx of inpatients with COVID-19 to the hospital to which the laboratory serves, there was a surge in demand of point of care tests (POCT) including arterial blood gases and glucose. To meet the increasing POCT workload, additional instruments, were procured and installed on an urgent basis. Alongside the POCT operator trainings were conducted on ad hoc basis and validation requirements were also fulfilled in a timely manner by the POCT team.

On the other hand, the laboratory also had to cope with the shortage of Trop-I kit, a vital biomarker, due to shipment delay and a few kits were borrowed from other sources until the receipt of the next shipment. Furthermore, the lab had to discontinue provision of another special biochemistry test, Immunoglobulin G4 (IgG4), due to the shortage because of delays in production and shipment of supplies from the manufacturer in United Kingdom, one of the countries hardest hit by the COVID-19 crisis.

#### 1.1.5 Impact on external quality assurance and accreditation requirements due to airspace closure

Due to logistic challenges Proficiency testing surveys From CAP were also not able to meet the timelines and the CAP directed the participating labs to perform and document alternate assessment testing by either split sample analysis, clinical correlation studies or direct observation of technique dependent tests as appropriate [20]. The quality assurance group in liaison with the sectional chemical pathologists came up with a plan swiftly and it was implemented to ensure the lab continues to deliver the highest standards of quality. Furthermore, the European Research Network for evaluation and improvement of screening, Diagnosis and treatment of inherited disorders of metabolism (ERNDIM), an external quality assurance scheme for the biochemical genetics laboratory also extended their dates of result submission, amid the airspace closure, which further eased the path for maintaining compliance with this scheme. At this instant, again having the privilege of having a virtual quality control platform in shape of Bio-Rad Unity Real Time, which allows for external peer group analysis in a networkable quality control program proved to be beneficial for the laboratory and was utilized efficiently.

Due to limited availability of testing kits and consumables, scheduled method validation/verification activities were also disrupted. The laboratory was preparing for an upcoming accreditation compliance audit by CAP in the later part of 2020, however as most audits, most of which requires physical presence of inspectors were called off by CAP due to the COVID-19 crisis, the status of the upcoming event also became uncertain.

#### 1.1.6 New tests introduction process halted

In order to ensure continuous growth, our lab core group plans introduction of new tests into the system keeping in sight the productivity, demand and regulatory approvals after appropriate protocols. For the current year, the laboratory had completed the required task associated with the new tests it was bound to offer including Anti-Phospholipase A2 Receptor antibodies (PLA2R) and acylcarnitine on its newly acquired tandem mass spectrometry platform. However, due to logistic issues the supply of reagents and consumables was either slowed down or halted. This further negatively impacted the anticipated revenue growth.

### 1.2 Processes Re-Defined

#### 1.2.1 Management of patient influx at the main laboratory

As the laboratory caters to both outpatients and inpatients, a noteworthy threat was the local spread of infection from un-screened patients and attendant visiting the facility for diagnostic workup [21,22]. A special counter was set up with appropriate safety protocol outside the main lab premises for screening using a standard questionnaire. Suspected COVID-19 cases were refrained from entering the laboratory premises and were diverted to the hospital's specially designated area for screening and management of such cases with appropriate protocol. As COVID-19 is transmitted by droplets and close contacts, patients, staffs and visitors to the laboratory had to wear mandatory masks and were provided with hand sanitizers at the counter and movement in common corridors were restricted.

#### 1.2.2 Internal surveillance measures

All healthcare professionals serving in the laboratory were strictly made to wear surgical masks and gloves as per the safety guidelines and safety officers were held responsible to ensure compliance [23]. Staff who developed symptoms were asked to report immediately and were given medical consultation within the hospital staff clinic. This enabled symptomatic staff to be identified promptly. Furthermore, the organization announced that all staff and their dependents, in case, if tested positive, their complete care will be fully covered financially by the hospital. This was re-assuring and taken positively by all the staff. Additionally, refresher training on the use of PPE was also conducted.

#### 1.2.3 Updates to the existing policies

Following good laboratory practices, all samples received at the section of clinical chemistry were regarded as infectious whether or not coming from suspected or confirmed cases and hazardous risk was minimized by reducing aerosolization, spill prevention and reducing unnecessary sample handling and movement. A fume hood, on war footing, was added to the processing bench to avoid aerosol and droplet spread specially for fecal samples. For provocative tests e.g. sweat chloride test and hydrogen breath test the technical staff was required to wear N95 mask, face shields, gowns (with sleeves) and gloves. Following sample collection, the procedure room was disinfected with hypochlorite and left vacant for at least 60 min for air changes before the next procedure.

#### 1.2.4 Housekeeping activities re-scheduled

Housekeeping cleaning schedules were re-defined and high exposure areas like the toilets, lifts, desks, tables were cleaned and wiped down several times a day with 5% Hypochlorite and alcohol-based sanitizers as appropriate. The premises were mopped three times a day with disinfectant. Discarded PPE and other clinical waste were collected in biohazard bags and disposed in a proper manner. The sectional safety officer was assigned the task of compliance monitoring.

#### 1.2.5 Continuing medical education (CME) via virtual platforms

An integral component of our daily work-based learning is dissemination of new knowledge via one to one lecture by faculty and senior managers for technologists, post graduate trainees and trainee technologists, case-based discussion, instrumentation module for resident and journal clubs. To foster teaching and learning environment and continue the routine teaching-learning activities the Section transitioned from face to face teaching sessions to online teaching activities. Virtual Learning Environments were explored by the faculty and all live lectures and Journal Clubs were being conducted via Microsoft Teams for synchronous teaching with the provision of recording for those who miss it. After getting familiar with Microsoft Teams. The Section has been conducting all administrative and educational meetings on Microsoft Teams or even ZOOM since the crisis started.

The faculty was formally trained to operate these software's by the information technology department and further utilized as trainers for residents and other staff. Feedback from the residents and technologists on the use of virtual podiums was positive as they can efficiently participate from their desk via their cell phones alongside the performance of other tasks assigned rather than scheduled events requiring their physical presence.

#### 1.2.6 Staff mental wellbeing measures

Most of the staff were demoralized by the pay cut and abandonment of appraisals, however they were encouraged by the leadership that the positive aspect is their jobs are maintained with provision of free health care amid the times of the crisis. Cookies with a thank you note were distributed to the staff to create recognition. Furthermore, the management was reinforced to ensure easily accessible channels for staff feedback and concerns. This COVID pandemic is generating significant stress and anxiety in our laboratory personnel particularly in those with existing mental health problems, in those with elderly or infants at home and in those with chronic disease. It was essential for all laboratory personnel, seniors and junior staff, to remain connected throughout this crisis. One of the main reasons for stress came from a feeling of uncertainty. As fear and uncertainty continued, the only key was to spread positivity by staying connected [24,25]. An important stress releaser activity at workplace is social networking with peers especially during intervals and time offs. Initially our dedicated dining room's seating arrangements were not in compliance as adequate distance was not maintained. However, to keep the staff connected and ensure relaxation time temporary arrangements were made to split the seating and staff's interval schedules were redefined to ensure compliance with social distancing.

### 1.3 Preparations for the post Covid-19 scenario

Most organization worldwide, including the Pakistani masses were not prepared to face a looming threat of a COVID‐19 pandemic. The preparations for such a crisis never existed in the books of laboratories especially for resource constrained setups in developing world where most focus is on cost savings and revenue enhancement. While one cannot predict when the next major pandemic will occur. However, the results of the timely measures highlighted above will be more accurately evident in the times to come, a substantial success was achieved in terms of infection control as of today, and none of our staff was tested positive during the outbreak, despite handling COVID positive samples. A few staff members were quarantined as part of the internal surveillance measures due to clinical suspicion or contact but their tests also turned negative and they resumed work after appropriate interval. A few salient lessons learnt from the crisis, which will enlighten the pathway for adequate workflow of the laboratory in the post COVID-19 world are summarized as follows.

• In times of such crisis, follow a proactive approach in anticipating and planning for the impact. Crisis response teams should be part of the regular laboratory groups with appropriate training. If need arises, prior standard operating procedures addressing the challenges the lab faced during the pandemic should be in place for immediate referral.
• The management, faculty and key stake holder should engage and coordinate formulating policies to tackle such emergency situations. The sectional leadership should pay special attention in identifying the “bottlenecks” in their respective domains for appropriate alternate plans.
• Decrease workload where possible in anticipation of depletion of resources. Built infrastructure for home phlebotomy and sample collection to facilitate the patients in lock downs and prevent unnecessary movement and reduce probability of exposure in the laboratory phlebotomy stations.
• Establish staff segregation either spatially or temporally, work from teams to be encouraged in order to have a few back up personnel if the front liners are exposed and need to be isolated.
• Staff training on the use of virtual connectivity podiums e.g. Microsoft Teams and ZOOM should be part of the regular training regimen at the entry level.
• It may be wise for educational institutes to encourage faculty to spend some of their time teaching online to gain experience and prepare themselves.
• As lack of human interaction will be the new norm in the post pandemic scenario, work culture will need modification on the grounds of social distancing. All meetings and consultations are to be conducted online with maximum participation. Social activities at the workplace including celebration parties, leisure breaks are to be halted.
• Rescheduling the tea and meal breaks for the staff in shifts, segregating the employs meal area with provision of limited seating and facility of prepacked meals ensured safety precautions are compliant.
• Salary cuts, postponed appraisals and the looming threat of losing their jobs created a negative impact on already disturbed mental status for most employs faced with painstaking objectors potentially risking the lives of their families when undertaking duties in a contagious outbreak. The lack of social activities further overburdened the scenario. Managers as effective counsellors should play a role and keep the morale high for effective functioning. Furthermore, the budget allocation should be diverted as such to ensure better incentives and remuneration; and may be fairer to give preference to groups that are more vulnerable.
• Post pandemic world with the anticipated overwhelming financial crisis, will require major resource allocation decisions for inventory management and prioritizing needs for new developments and projects.

The post COVID lab functioning was evidently different from the normal scenario. Even though, the lab continued to serve 24/7, in all three shifts as prior; but the number of staff was reduced to minimize exposure. A significant time of the daily work place activities of Clinical Chemistry faculty and managerial staff is spent in quality control & quality assurance activities, administrative meetings, internal and external audits, teaching sessions, grand rounds, journal clubs etc. requiring physical interaction and interventions. However, amid the social distancing measures all such activities were transformed to virtual sessions, including a few temporary postponements. Additionally, most of the staff were trained on using virtual platforms using self-directed learning, which is the new norm and essential skill for the post COVID era.

Additionally, a literature review of approaches adopted by clinical laboratories worldwide during the pandemic, revealed a report from a United States of America based cytology laboratory, which implemented likewise measures specifically pertaining to social distancing, revision of certain policies, safety measures, re-evaluation of staffing needs, online meetings and virtual academic activities [26].

Taken together, many questions remain for this emerging infection, the take home message derived is that a few of yesterday's practices and policies may be too old or irrelevant in the post COVID-19 laboratory. As social distancing will be the new normal, a potential impact may be seen in an intensification of digital infrastructure of the laboratories, to allow few of the stakeholders to effectively function remotely in compliance with the regulatory and accreditation requirements [27]. New ways of ensuring cost cuttings and reaching a break-even point at minimum has to be formulated, keeping the interests and security of your frontline warriors in sight.

## 2 Conclusion

With the current state of local spread in the country and amid the high chances of contracting COVID-19, it is inevitable that the clinical laboratories take drastic measures and succumb to acceptable alternate plans for ensuring the safety and interests of its valuable employs alongside continuousness of provision of diagnostic services for better health outcomes, in times of the pandemic. We must be prepared to adapt to the constantly evolving phases and emergent scenarios during the pandemic with a proactive approach. We hope that other clinical chemistry laboratories globally will benefit from our experience in dealing with the COVID-19 crisis. In 2020, we woke up in a different world, with different set of rules; for work environment, financial priorities and social norms, both at home and workplace. The earlier we adapt and embrace the change, the earlier we will be able to create a safe, workable, financial, social, environment for ourselves, our colleagues and families.

## Provenance and peer review

Not commissioned, externally peer reviewed.

## Ethical approval

Not required as it is a narrative review and does not include any human data or intervention.

## Sources of funding

None.

## Author contribution

SA performed the majority of the writing work in the first draft. LJ helped in designing the outline and the writing work. HM helped with references search and retrieval and gave suggestions to improve the first draft. AHK and FG did the critically revisions of the manuscript. IS conceived the idea and coordinated the writing of the paper and reviewed the final draft. All authors have reviewed the final draft and agreed upon.

## Consent

Not required.

## Registration of research studies

Name of the registry: Not requiredUnique Identifying number or registration ID: Not requiredHyperlink to your specific registration (must be publicly accessible and will be checked):

## Guarantor

Dr. Imran Siddiqui, Professor, Department of Pathology and Laboratory Medicine, Aga Khan University Hospital, Stadium Road, Karachi, Pakistan.P.O. Box 3500, Telephone: 021–34861927. Email: imran.siddiqui@aku.edu.

## Declaration of competing interest

None.
